# Transcriptome Analysis Reveals Altered Expression of Genes Involved in Hypoxia, Inflammation and Immune Regulation in *Pdcd10*-Depleted Mouse Endothelial Cells

**DOI:** 10.3390/genes13060961

**Published:** 2022-05-27

**Authors:** Carmela Fusco, Grazia Nardella, Lucio Di Filippo, Elisabetta Dejana, Davide Cacchiarelli, Antonio Petracca, Lucia Micale, Matteo Malinverno, Marco Castori

**Affiliations:** 1 Division of Medical Genetics, Fondazione IRCCS-Casa Sollievo della Sofferenza, 71013 Foggia, Italy; g.nardella@operapadrepio.it (G.N.); a.petracca@operapadrepio.it (A.P.); l.micale@operapadrepio.it (L.M.); m.castori@operapadrepio.it (M.C.); 2 Next Generation Diagnostic s.r.l., 80078 Pozzuoli, Italy; lucio.difilippo@ngdx.eu; 3 Vascular Biology Unit, FIRC Institute of Molecular Oncology Foundation (IFOM), 20139 Milan, Italy; elisabetta.dejana@ifom.eu (E.D.); matteo.malinverno@ifom.eu (M.M.); 4 Armenise/Harvard Laboratory of Integrative Genomics, Telethon Institute of Genetics and Medicine (TIGEM), 80078 Pozzuoli, Italy; d.cacchiarelli@tigem.it; 5 Department of Translational Medicine, University of Naples “Federico II”, 80126 Naples, Italy; 6 School for Advanced Studies, Genomics and Experimental Medicine Program, University of Naples “Federico II”, 80126 Naples, Italy

**Keywords:** *Pdcd10*, cerebral cavernous malformation, transcriptomic analysis, hypoxia, HIF-1signaling, inflammation, immune response

## Abstract

Cerebral cavernous malformations (CCM) are capillary malformations affecting the central nervous system and commonly present with headaches, epilepsy and stroke. Treatment of CCM is symptomatic, and its prevention is limited. CCM are often sporadic but sometimes may be multifocal and/or affect multiple family members. Heterozygous pathogenic variants in *PDCD10* cause the rarest and apparently most severe genetic variant of familial CCM. We carried out an RNA-Seq and a Q-PCR validation analysis in *Pdcd10*-silenced and wild-type mouse endothelial cells in order to better elucidate CCM molecular pathogenesis. Ninety-four differentially expressed genes presented an FDR-corrected *p*-value < 0.05. A functionally clustered dendrogram showed that differentially expressed genes cluster in cell proliferation, oxidative stress, vascular processes and immune response gene-ontology functions. Among differentially expressed genes, the major cluster fell in signaling related to inflammation and pathogen recognition, including HIF1α and Nos2 signaling and immune regulation. Validation analysis performed on wild-type, Pdcd10-null and Pdcd10-null reconstituted cell lines was consistent with RNA-Seq data. This work confirmed previous mouse transcriptomic data in endothelial cells, which are recognized as a critical tissue for CCM formation and expands the potential molecular signatures of *PDCD10*-related familial CCM to alterations in inflammation and pathogen recognition pathways.

## 1. Introduction

Cerebral cavernous malformations (CCM) are common vascular malformations derived from capillaries and small vessels of the central nervous system (CNS) [1]. Major clinical manifestations include intracranial haemorrhage, seizures and headache. Given the clinical unpredictability of CCM, surgery, stereotactic radiosurgery, pain medications and pharmacological prevention of seizures are the only therapeutic resources after neuroimaging detection of an otherwise unexpected lesion or, more commonly, after abrupt or subacute manifestations. Disease prevalence is estimated at 0.16–0.5% in the general population and often occurs sporadically [2]. More rarely, CCM may be multifocal and/or aggregate in families (familial CCM—FCCM) [3]. FCCM are caused by heterozygous, deleterious variants in either one of three genes encoding for interacting proteins, comprising Krev1 Interaction Trapped 1 (*KRIT1*; *CCM1;* MIM#604214), Malcavernin (alias *MGC4607*; *CCM2*; MIM#607929) and Programmed Cell Death 10 (*PDCD10*; *CCM3*; MIM#609118). Loss-of function is the prevalent molecular mechanism in FCCM. Genotype–phenotype correlations in FCCM are poor, and molecular data have limited clinical applications to date. More recently, the identification of a deleterious variant in either one of the known genes was considered mandatory for clinical trial enrolment in FCCM [4]. A better understanding of the biological diversity underpinning clinical variability in FCCM will improve prognostication, management planning and treatment approaches for future patients.

Alterations of *PDCD10* are the rarest genetic cause of FCCM and tend to associate with a more aggressive phenotype with an earlier age of onset [5]. The encoded protein is identified as a key molecule for intracranial angiogenesis and endothelial cell homeostasis in both in vitro studies and animal disease models. In particular, studies in isolated endothelial cells show that *Pdcd10*-mediated pathways include Notch signaling, VEGF signaling and the ERK/MAPK pathway [6,7]. Zebrafish models reveal that Pdcd10 plays an essential role in early embryonic angiogenesis and cardiovascular development [8,9,10,11]. Furthermore, the murine *Pdcd10* model shows that the Pdcd10 protein takes part in different intracellular signaling, which affects cell junction, apoptosis and stress responses [12]. Despite the many collected biochemical in vitro and in vivo data on *PDCD10*, the molecular pathogenesis of *PDCD10*-related FCCM remains only partially understood, and this lack of knowledge impacts the development of tailored patient’s management.

Here, we explored the consequences of *Pdcd10* silencing in mouse endothelial cells (ECs) by employing a transcriptomic analysis. This study allowed us to identify novel *Pdcd10*-controlled molecular pathways and offered the possibility of providing novel insights into FCCM pathogenesis and therapeutic targets.

## 2. Materials and Methods

### 2.1. Cell Lines

An immortalized mouse aortic EC line was generously gifted by Prof. Francesca Boccafoschi (Health Science Department, University of Piemonte Orientale, Novara, Italy). Cells were cultured in D-MEM with Glutamax supplemented with 20% FBS, 1% penicillin (100 U/mL) and streptomycin (100 μg/mL) (Thermo Fisher Scientific, Waltham, MA, USA) and grown in a 5% CO_2_ incubator at 37 °C. For validation studies, immortalized mouse lung-derived endothelial cell lines of either wild-type or knocked out for *Pdcd10* (here named as EC-Ctrl*, Pdcd10*iEC-KO, respectively) and endothelial cell lines from *Pdcd10* knockout mice, to which the human PDCD10 (here named as *Pdcd10iEC-KO^+Pdcd10^*) were re-added, were cultured as described in [13]. In brief, to generate Pdcd10^−/−^ cells re-expressing mGFP-tagged PDCD10, Pdcd10^−/−^ were transduced with the recombinant lentivirus Lenti ORF clone mGFP-tagged PDCD10 (OriGene Technologies Inc., Rockville, MD, USA). The human PDCD10 aminoacid sequence presents a single substitution (p.V192I) compared to the Pdcd10 mouse protein. The Lenti ORF clone mGFP-tagged PDCD10 vector was already used in mouse cells as reported in [13]. The recombinant lentiviruses were resuspended in serum-free MCDB-131 medium and added to the cells for 1 h at 37 °C. To increase the number of the cells, the cells were then passaged four times.

### 2.2. RNA Interference

Stealth RNAi duplexes designed against *Pdcd10* (Thermo Fisher Scientific, Waltham, MA, USA) or stealth RNAi negative control (Thermo Fisher Scientific, Waltham, MA, USA) were transfected in EC cells (here named as si*Pdcd10*-ECs and siCNT-ECs, respectively) using Lipofectamine RNAiMAX (Thermo Fisher Scientific, Waltham, MA, USA) and according to the manufacturer’s protocol. 

### 2.3. Western Blotting

The EC line was plated in six-well culture dishes at a density of 1 × 105 cells/mL and then transfected with the indicated Stealth RNAi duplexes. After 48 h, cells were lysed in 1x D-PBS, 0.025% NP-40 and protease- and phospho-inhibitors (Roche, Pasadena, CA, USA). Total cell lysates were analyzed by 10% SDS-PAGE page electrophoresis, transferred to nitrocellulose membrane and blotted with anti-Pdcd10 (Proteintech Cat#10294-2-AP, RRID: AB_2162153) and anti-β-Actin (Santa Cruz Biotechnology Cat#sc-47778 HRP, RRID:AB_2714189) [14,15,16] antibodies. The specificity of the anti-Pdcd10 antibody was determined through Pdcd10 silencing by comparing the control and silenced cell line. Horseradish peroxidase-conjugated anti-rabbit Ab (Bio-Rad Cat# 1706515, RRID:AB_2617112) was used as a secondary antibody [17,18].

### 2.4. RNA Extraction

Total RNA was extracted using a mini RNase kit reagent (Qiagen, Hilden, Germany). The quality of nucleic acids was assessed using Nanodrop ND1000 (EuroClone, Milan, Italy). The RNA quantity was evaluated by Qubit RNA BR Assay Kit (Thermo Fisher Scientific, Waltham, MA, USA). The RNA integrity was assessed by the RNA Integrity Number (RIN) using the Agilent RNA 6000 Nano Kit on the BioAnalyzer 2100 (Agilent, Boulder, CO, USA). All analyzed samples displayed a RIN above 9.50.

### 2.5. Library Preparation

Total RNA of si*Pdcd10*-EC and siCNT-EC lines from three replicas of each cell type was quantified using the Qubit 2.0 fluorimetric Assay (Thermo Fisher Scientific, Waltham, MA, USA). A poly-A enriched library was generated with the TruSeq RNA-Seq Library Preparation Kit v2 (#RS-122-2001, Illumina, San Diego, CA, USA) according to the manufacturer’s instructions. Library quality control was performed using the Agilent 2100 Bioanalyzer. Indexed libraries were sequenced at the CRS4 Next Generation Sequencing facility with the HiSeq 3000 instrument to generate ~40 M 50 bp single-end reads per sample. Read and library quality was assessed by running FastQC (RRID:SCR_014583) and RSeQC (RRID:SCR_005275) [19] on FASTQ and aligned BAM generated with STAR. Transcript abundance was estimated with Kallisto [20], and differentially expressed genes (DEGs) were identified using DeSeq2 (RRID:SCR_015687) [21] R package with an FDR corrected *p*-value < 0.05. Enrichment analysis was performed with ToppCluster (RRID:SCR_001503) [22].

### 2.6. Quantitative PCR (qPCR)

Total RNA from si*Pdcd10*-EC and siCNT-EC and from *Pdcd10*iEC-KO, EC-Ctrl and *Pdcd10iEC-KO^+Pdcd10^* was reverse transcribed using the RT2 First Strand Kit (Qiagen, Hilden, Germany), according to the manufacturer’s instructions. Oligos for the quantitative real-time PCR (Q-PCR) were designed using the Primer express program (RRID:SCR_014326) [23] with default parameters (Table S1). *Gapdh* and *Actin* were used as reference genes. The reactions were run in triplicate in 10 μL of final volume with 10 ng of sample cDNA, 0.3 mM of each primer and 1XPower SYBR Green PCR Master Mix (Thermo Fisher Scientific-Applied Biosystems, Carlsbad, CA, USA). Reactions were set up in a 384-well plate format with a Biomeck 2000 (Beckmann Coulter, Carlsbad, CA, USA) and run in an ABI Prism7900HT (Thermo Fisher, Scientific-Applied Biosystems, Carlsbad, CA, USA) with default amplification conditions. Raw Ct values were obtained using SDS 2.4 (Applied Biosystems, Carlsbad, CA, USA). Calculations were carried out by the comparative Ct method as reported in [24]. Significance was determined by a two-tailed unpaired *t*-test for means [24].

### 2.7. Bioinformatics Workflow

The raw data were analyzed by Next Generation Diagnostics srl, which is the proprietary of the full-length mRNA-seq pipeline (v1.0) comprising quality control, alignment to the reference and counting steps [25]. The raw expression data were normalized, analyzed and visualized by Rosalind HyperScale architecture (RRID:SCR_006233) [21] (Hennig, C. Cran-package fpc. released on 6 December 2020 https://cran.r-project.org/web/packages/fpc/index.html) (OnRamp BioInformatics, Inc.; San Diego, CA, USA).

Clustering of genes for the final heatmap of differentially expressed genes was carried out using the PAM (Partitioning Around Medoids) method using the fpc R library (https://cran.r-project.org/web/packages/fpc/index.html published 6 December 2020). Enrichment analysis for Gene Onthology was conducted using the topGO package [26]. 

Several database sources were referenced for enrichment analysis, including Interpro, NCBI, MSigDB, REACTOME and WikiPathways. Enrichment was calculated relative to a set of background genes relevant to the experiment. The top 50 biological process terms for Elim *p*-value were analyzed with Revigo [27].

All datasets sequencing data were deposited to Gene Expression Omnibus (GEO) (https://www.ncbi.nlm.nih.gov/geo/query/acc.cgi?acc=GSE186523; ID number: GSE186523 released on 1 October 2022). 

### 2.8. Confocal Microscopy

For immunocytochemical analysis, EC-Ctrl and *Pdcd10*iEC-KO cells were plated in 12-well culture dishes at a density of 1 × 55 cells/mL and then fixed in 4% paraformaldehyde and incubated with 0.5% Triton-X100 in phosphate-buffered saline for 1 h. After, the cells were counterstained with anti-PECAM1 antibody (1:50; 551,262; BD Pharmingen) for 2 h at room temperature, followed by incubation with Alexa Fluor goat anti-mouse IgG (1:500 dilution, #A11011 Thermo Fisher Scientific, Waltham, MA, USA), for 2 h at room temperature finally with 4,6-diamidino-2-phenylindole (DAPI, Molecular Probes, #D1306).

Confocal microscopy was performed using a confocal microscope (TCS SP5, Leica, Wetzlar, Germany), with the ImageJ software (NIH, New York, NY, USA) used for image analysis.

### 2.9. Statistical Analysis

Statistical analysis of immunoblotting and Q-PCR assays were performed using an unpaired, two-tailed Student’s *t*-test (Excel software) (* *p* < 0.05, ** *p* < 0.01).

## 3. Results

### 3.1. Pdcd10-Related Transcriptomic Profile

**Figure 1:**
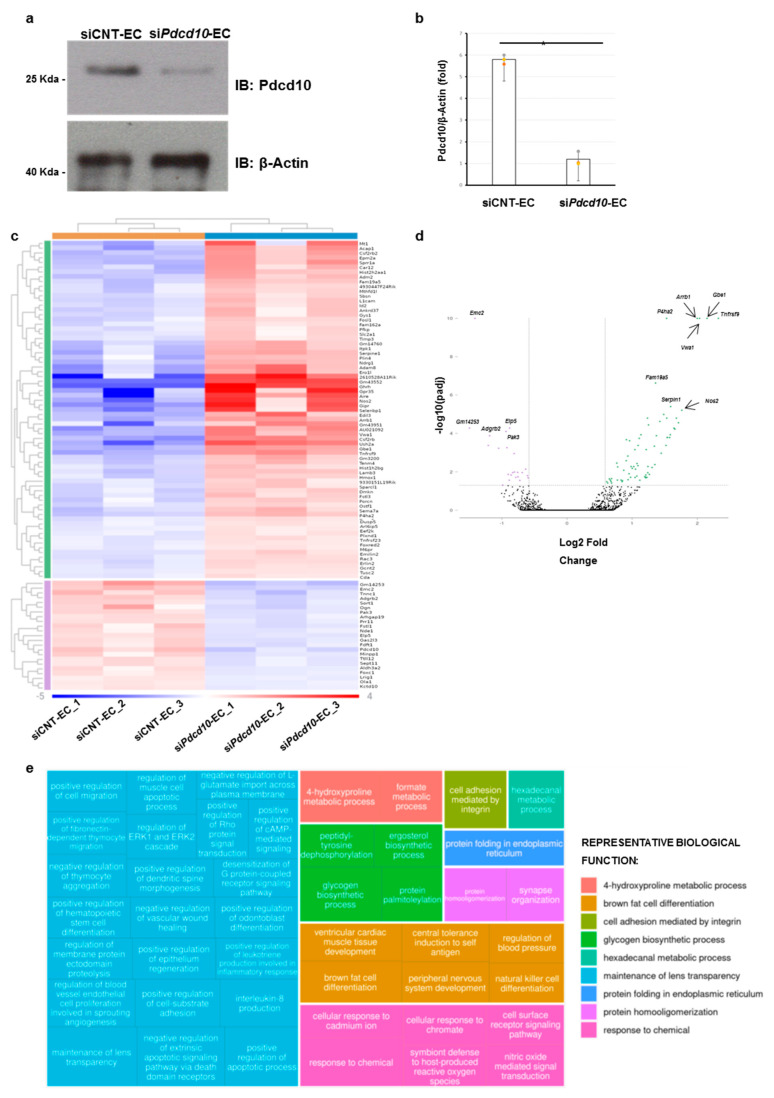
RNA-seq data. (**a**) Total lysates were obtained from si*Pdcd10*-ECs compared to siCNT-EC, separated on 10% SDS-gel and subjected to immunoblotting with indicated antibodies. (**b**) Relative levels of protein intensity related to Pdcd10/β-Actin was quantified by densitometry using Image J analysis software, and the mean of each quantification was reported in the graph. Graphs show averages calculated on three different biological experiments represented by three points (green, yellow and orange). Scale bars represent standard errors. Values are expressed as mean ± SEM (* *p* < 0.05, *n =* 3). (**c**) Heatmap of gene ontology enrichment analysis of functional differences between si*Pdcd10*-EC and siCNT-EC lines. The statistical significance in the heatmap was calculated and presented based on the -log10 false discovery rate (FDR) corrected *p*-values (blue indicates significant upregulated genes; red indicates significant downregulated genes). The colored scale bar below shows the color scaling with FDR values. The horizontal or vertical bars (violet, blue, orange and green) represented the different clusters of genes coming from a gene ontology analysis generated by Rosalind analysis. On the right-hand side of the Heatmap, a list of DEGs was reported. (**d**) Volcano plot showing the differentially expressed genes (violet points represent downregulated genes, green points represent upregulated genes, and the adjusted *p*-value threshold plotted on the Y-axis is 1.3). (**e**) Treemap representing over-represented biological functions, grouped into processes. Sizes of rectangles are proportional to the number of genes involved in a specific biological process. On the right of the Treemap, the more representative biological function for each cluster is indicated.

In order to identify novel molecular pathways potentially altered by *Pdcd10* silencing, we carried out RNA-sequencing (RNA-seq) analysis in wild-type (i.e., siCNT-EC) and *Pdcd10-silenced* lines (i.e., si*Pdcd10*-EC) from aortic murine immortalized ECs. An in vitro culture of mouse ECs were previously used for exploring the molecular pathogenesis of FCCM, as these cells can be considered the counterpart of human endothelial tissue [28]. We first silenced *Pdcd10* in the EC line by the transfection of specific Stealth RNAi for *Pdcd10*. We found a reduction of ~80% protein level in si*Pdcd10*-EC compared to siCNT-EC by Western blot assay (Figure 1a,b).

Transcriptome analysis revealed 94 DEGs with an adjusted *p*-value (pAdj) < 0.05. The expression of 94 DEGs differed significantly by at least 1.5-fold change (71 upregulated genes vs. 23 downregulated genes) (Figure 1c, Table 1 and Table S2). Figure 1d represent the volcano plot illustrating the 94 DEGs with a -log10(p-adj) threshold of at least 1.3. Among the most upregulated genes in si*Pdcd10*-EC, we identified the TNF Receptor Superfamily Member 9 gene (*Tnfrsf9*, Entrez Gene ID (E_ID): 21942; log fold change (LogFc) = 2.323), the 1,4-alpha-Glucan Branching Enzyme 1 (*Gbe1*, E_ID: 74185; LogFc = 2.15), the Arrestin beta 1 gene (*Arrb1*, E_ID: 109689; LogFc = 2.000), the Von Willebrand Factor A Domain Containing 1 gene (*Vwa1*, E_ID: 246228; LogFc = 2.035) and the Prolyl 4-Hydroxylase Subunit alpha 2 (*P4ha2*, E_ID: 18452; LogFc = 1.5289) mainly involved in cytokine and immunological systems. Among the downregulated genes, the ER Membrane Protein Complex Subunit 2 (*Emc2*, E_ID: 66736; LogFc = −1.413), the Adhesion G Protein-Coupled Receptor B2 (*Adgrb2*, E_ID: 230775; LogFc = −1.05) and the P21 (RAC1) Activated Kinase 3 (*Pak3*, E_ID: 18481; LogFc = −0.93717), which are predominantly associated with angiogenesis and immunological systems, showed the most significant values (Table 1).

### 3.2. Pathway Analysis of Differentially Expressed Genes

Gene set functional enrichment analysis using the Rosalind HyperScale web platform identified a number of differently represented biological functions. The top enriched gene ontology functions were related to the 4-hydroxyproline metabolic process, brown fat cell differentiation, cell adhesion mediated by integrin, glycongen biosynthetic process, hexadecanal metabolic process, maintenance of lens transparency, protein folding in the endoplasmic reticulum, protein homooligomeritation and response to chemical (Figure 1e).

**Figure 2:**
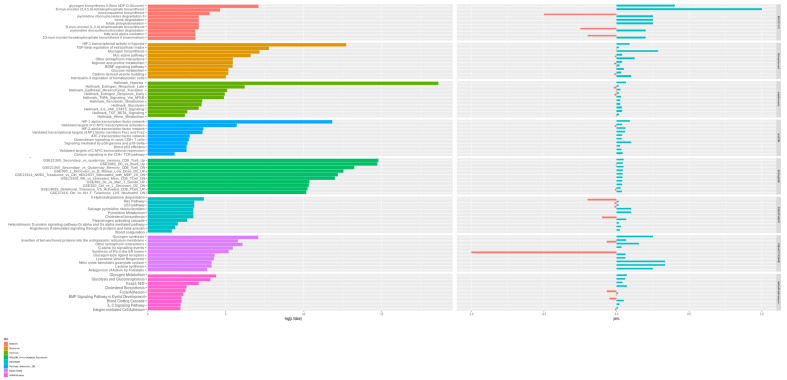
Enriched pathways. Bar plot (**left**) reporting the main significantly enriched biological processes extracted by different ReviGO databases, enrichment score > 3. Stacked bar plot (**right**) accounting for proportions of upregulated and downregulated genes for each biological process. The top 10 pathways by *p*-value were extracted from the main databases. For each pathway, the percentage of significant genes contained in the term was calculated.

Enrichment pathway analysis identified 21 significantly enriched pathways (Figure 2, Table 2 and Table S3). The most significantly altered pathways involve hypoxia (p-Adj = 3.0 × 10^−7^, associated with 13 DEGs), HIF-1α transcriptional activity (p-Adj = 0.00020, associated with 6 DEGs), Nod2 signaling (p-Adj = 0.00352, associated with 8 DEGs) and selected immunological signatures related to the memory CD8 T-cells (p-Adj = 0.00071, associated with 9 DEGs), IL12-CD8 associated T-cells (p-Adj = 0.01271, associated with 7 DEGs) and the dendritic cell-elicited B-cells activation (p-Adj = 0.04286, associated with 6 DEGs) (Figure 2, Table 2, Table 3 and Table S3).

Among the other significant enrichment pathways there are glycogen biosynthesis (p-Adj = 0.01995, associated with 2 DEGs), estrogen response (p-Adj = 0.03626, associated with 7 DEGs), endothelial-to-mesenchymal transition (End-MT, p-Adj = 0.05306, associated with 6 DEGs) and TNFα signaling (p-Adj = 0.05306, associated with 6 DEGs) (Figure 2, Table 2 and Table S3).

### 3.3. Validation Study of Differentially Expressed Genes in Mouse Endothelial Cells

**Figure 3:**
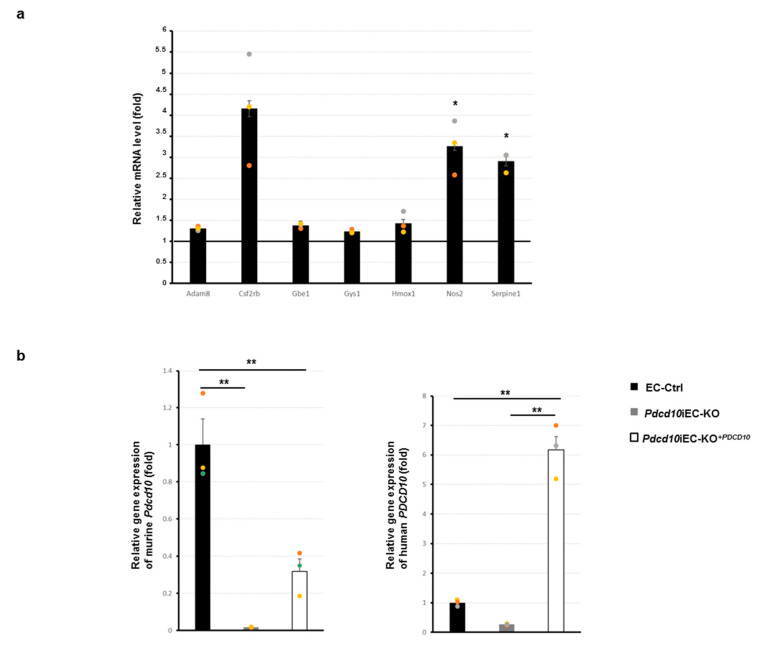

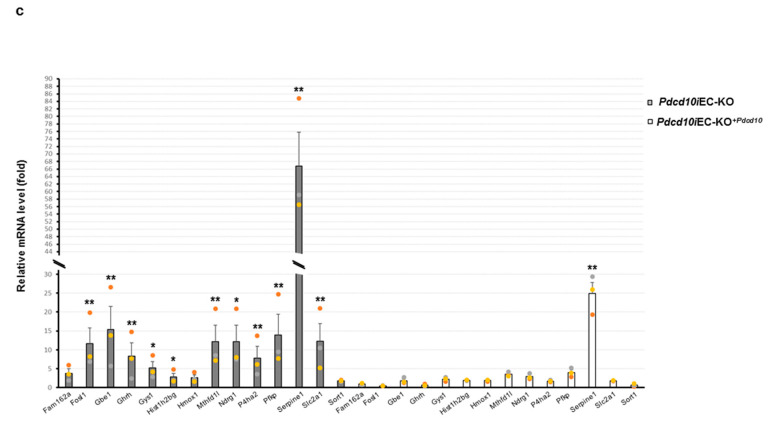
RNA-seq validation analysis. (**a**) Q-PCR results of a set of DEGs in siPdcd10-ECs and siCNT-EC. The fold change value relates to the mean expression levels of siCNT-EC, which were set as value 1. The mean expression levels of siCNT-EC derived from three biological replicates; each of these was run in three technical replicates. Graphs show averages calculated on three different biological experiments represented by three points (green, yellow and orange); each point characterized the mean of three technical replicates. Error bars represent standard errors. Values are expressed as mean +/− SEM (** *p* < 0.01, *n* = 3). (**b**) Q-PCR of Pdcd10 expression level of mouse (left) and human (right) gene results came from Pdcd10iEC-KO, EC-Ctrl, and Pdcd10iEC-KO^+Pdcd10^ cell lines. (**c**) Q-PCR of some DEGs came from Pdcd10iEC-KO, EC-Ctrl and Pdcd10iEC-KO^+Pdcd10^ lines were reported. Graphs show averages calculated on three different biological experiments represented by three points (green, yellow and orange); each point characterized the mean of three technical replicates. The fold change value relates to the mean expression levels of EC-Ctrl, which were set as value 1. The mean expression levels of EC-Ctrl derived from three biological replicates; each of these was run in three technical replicates. Values are expressed as mean +/− SEM (** *p* < 0.01, * *p* < 0.05, *n* = 3).

To validate the transcriptomic data, we performed Q-PCR analysis in the lung-derived si*Pdcd10*-EC line. We confirmed the upregulation of a set of genes prioritized based on their functional classification that was significantly perturbed in *siPdcd10-EC* lines, including ADAM Metallopeptidase Domain 8 *(Adam8*), Colony Stimulating Factor 2 Receptor Subunit beta(*Csf2rb)*, *Gbe1*, Glycogen Synthase 1 (*Gys1*), Heme Oxygenase 1 (*Hmox1*), Nitric Oxide Synthase 2 (*Nos2*) and Serpin Family E Member 1 (*Serpin1*), compared to control cell line (Figure 3a). *Csf2rb*, *Hmox1*, *Nos2* and *Serpin1* resulted the most upregulated genes. Transcriptome data were also validated by an independent Q-PCR assay performed on mRNA from either *Pdcd10*iEC-KO and EC-Ctrl lines and from *Pdcd10iEC-KO^+Pdcd10^*, in which the human PDCD10 was over-expressed [13] (Figure 3b). Through analysis of different biological pathways databases, we selected a set of the most representative biological processes (Table S3). Firstly, we stained the *Pdcd10*iEC-KO and EC-Ctrl cells with the endothelial cell marker PECAM1 in order to verify the endothelial profile (Supplementary Figure S1). Then, we measured the expression of a set of DEGs associated with the main significant deregulated pathways, including hypoxia, HIF-1α, NOD2 and immunological-associated signaling, for which the functional association with *PDCD10/Pdcd10* has not been established yet. We showed an upregulation of all tested genes in *Pdcd10*iEC-KO compared with EC-Ctrl lines, of which 11 resulted upregulated. Furthermore, we also addressed a rescue by a reduction of gene expression in *Pdcd10iEC-KO^+Pdcd10^* cells (Figure 3c). Among them, *Serpin 1* resulted more upregulated than the other analyzed genes. 

## 4. Discussion

Here, we carried out a transcriptome profiling analysis in mouse endothelial *Pdcd10* silenced cells and validated our findings in ECs obtained from *Pdcd10* knockdown mice and from *Pdcd10* knockdown mice re-expressing the human *PDCD10* in a subset of selected genes by choosing the genes associated with enriched signaling. Novel findings included pathway alterations of hypoxia, HIF-1α, NOD2 signaling, specific immunological pathways, glycogen biosynthesis, End-MT and TNFα signaling. 

*PDCD10* encodes for an evolutionarily conserved protein physiologically involved in different intracellular signaling pathways such as cell junction, angiogenesis, apoptosis, End-MT and stress responses [12,28]. PDCD10 is highly expressed in the neurovascular unit, and this explains the organ-specific manifestations of FCCM due to heterozygous loss-of-function variants in *PDCD10*. While current management of FCCM is symptomatic, the growing insights into the FCCM molecular pathogenesis are opening the path to innovative therapies aimed at preventing complications. From this perspective, there are two drug-repurposing clinical trials exploring the efficacy of propranolol and atorvastatin in reducing disease manifestations in adults with CCM [4,29]. Hopefully, a deeper understanding of the subcellular and cellular mechanisms leading to CCM formation and rupture in FCCM will ease the identification of further candidate targets for known and novel molecules. 

In order to highlight novel potential genetic targets, several transcriptomic studies related to both coding and noncoding RNA were conducted on CCM patients’ tissues without molecular characterization [30,31,32,33]. These studies showed dysregulation of several signaling which clustered in neuronal activity, angiogenesis, extracellular matrix signaling and vascular integrity. Abou-Fadel and co-authors provided a combination of proteomic and transcriptomic analysis from silencing *CCM* genes in endothelial cells and from *Ccm1* and *Ccm2*-knockout zebrafish embryos, revealing a unique portrait detailing alterations in angiogenesis and endothelial permeability [34]. 

To date, three RNA-Seq analyses aiming to profile the molecular role of PDCD10 in CCM pathogenesis were reported. The first one consisted of a transcriptomic study from brain lesions of *Pdcd10* knockdown mice and identified alterations in neurological signal transduction, postsynaptic signaling and oxidative stress [35]. A combination of transcriptomic analysis derived from mouse and C. elegans endothelial *Pdcd10*-silenced cells revealed a set of genes related to integrin-signaling and vesicle transportation [36]. Recently, Orsenigo and co-authors reported an in-depth single-cell RNA sequencing in a Pdcd10-mouse model mapping the transcriptional diversity of endothelial cells in vascular lesions [37]. The amount of transcriptomic data reported, if confirmed and accurately validated in other cell lines and/or disease models, will surely stimulate the development of novel therapeutic strategies.

In the present study, we first confirmed alterations in pathways identified as abnormal in previous RNA-Seq studies in different tissues and including oxidative stress, integrin-signaling, vesicle transportation, angiogenesis and vascular integrity [28,36,37,38,39]. Our investigations also identified the involvement of novel pathways, including hypoxia and HIF-1α signaling, *NOD2*-related pathway and immune response. 

### 4.1. Hypoxia and HIF-1α Signaling

Many DEGs in this study were related to the hypoxia regulatory network, which is one of the most crucial pathways implicated in the control of the immune response, tissue homeostasis and endothelial signaling in the vasculature. HIF-1α is the key regulator of tissue response to hypoxia [40]. HIF-1α is critical for the development of atherosclerosis through cell-specific responses by acting on endothelial cells, vascular smooth muscle cells and macrophages. HIF-1α controls different pathophysiological processes, including vascular dysfunction, atherosclerosis, myocardial infarction and stroke. In our study, DEGs with at least a 1.5-fold increase in expression linked to hypoxia included cytokines/growth factors (N-Myc Downstream Regulated 1 (*Ndrg1*), *Hmox1*, Inhibitor of DNA Binding 2 (*Id2*), *Family With Sequence Similarity 162 Member A* (*Fam162A*), Solute Carrier Family 2 Member 1 (*Slc2a1*)), receptors (*Gbe*, *Gys1*) and other signaling proteins (*Serpine1*, *Nos2*, Solute Carrier Family 2 Member 1 (*Slca1*), Selenium Binding Protein 1 (*Selenbp1*), Phosphofructokinase, Platelet (*Pfkp*), Endoplasmic Reticulum Oxidoreductase 1alpha (*Ero1l*), Prolyl 4-Hydroxylase Subunit alpha2 (*P4ha2*), Carbonic Anhydrase 12 (*Car12*), *Gys1*, *Fam162A*, and Glucosaminyl (N-Acetyl) Transferase 2 (*Gcnt2*)). Among them, *Serpine1*, which encodes for a member of the serine proteinase inhibitor superfamily, is interesting as it contributes to innate antiviral immunity, and its expression is influenced by HIF-1α as a result of stimulation of cellular migration and cell-adhesion markers expression. Both these mechanisms, if altered, might affect permeability, which appears defective in FCCM patients’ cell lines [41]. 

### 4.2. NOD2 Signaling

Our transcriptomic data also reported a significant transcriptional activation of *Nod2*-associated genes. NOD2/Nod2 is an intracellular pattern recognition receptor that stimulates the host immune response. A variety of extracellular stimuli can activate distinct signaling pathways that converge to initiate *NOD2/Nod2* expression. Specific cell wall components of bacteria and fungi can trigger the innate immune signaling cascade and then lead to *NOD2/Nod2* expression. Following activation, NOD2/Nod2 stimulates pro-inflammatory pathways such as NF-κB and MAPK signaling [42] and thereby contributes to host defence via the production of inflammatory cytokines, antimicrobial molecules [43] and mucins [44]. More specifically, NOD2/Nod2 acts as an immune sensor in the gut microbiota balance and the related microbiota–host interaction. Research into the role of the gut microbiome in modulating brain function has rapidly increased over the past 10 years. Increasing clinical and preclinical evidence implicates the microbiome as a possible key susceptibility factor for neurological disorders, such as Alzheimer’s disease, autism spectrum disorder, multiple sclerosis, Parkinson’s disease and stroke [45]. Interestingly, a recent study showed that CCM lesions arise from an excess of MEKK3 signaling downstream of TLR4 stimulation by the gut microbiome. This suggests the existence of a gut–brain disease axis in FCCM [46,47].

We demonstrated dysregulation of several genes which converge to NOD2/Nod2 signaling and include *Csfr2b*, *Ndrg1*, *Car12*, *Csf2rb2*, Semaphoring A7 (*SemaA7*), *Fam162A* and *Slc2a1* [48,49,50,51]. In light of the recent discoveries of a possible role of the microbiota in the pathogenesis of CCM, our preliminary findings could be interpreted as a link between CCM formation and altered gut-microbiota interactions via Nod2 pathway dysfunction in *PDCD10*-related FCCM.

### 4.3. Immunological Signatures

Human T cells, CD4^+^ T and CD8^+^ T cells coordinate adaptive immune responses and are essential for establishing protective immunity and maintaining immune homeostasis through the production of cytokines and effector molecules. CD4^+^ T cells secrete cytokines to recruit and activate other immune cells, while CD8^+^ T cells acquire cytotoxic functions to directly kill infected cells [52]. The CNS is recognized as immune-privileged. However, recent advances highlight interactions between the peripheral immune system and CNS in controlling infections and tissue homeostasis [53,54]. One study suggested the role of inflammation in the CCM pathogenesis by revealing a robust inflammatory cell infiltration in CCM [55]. In our work, DEG analysis identified genes involved in the immune and inflammation response, such as *Adam8*, *Gys1* and Elastin Microfibril Interfacer 2 (*Emilin2*). 

ADAM8 was described as a promoter of macrophage infiltration upon inflammation [56]. GYS1 might be a novel therapeutic strategy for chronic inflammatory arthritis since its expression deregulation was associated with chronic inflammation in patient cell lines [57]. Finally, EMILIN2 stimulates the production of a number of cytokines involved in angiogenesis and inflammation [58]. Overall, the significant overexpression of these genes in our study suggests a relationship between *Pdcd10* expression and the immune and inflammatory responses. These findings suggest that the immunological profile may be closely implicated in the CCM pathogenesis at least in *PDCD10*-related FCCM.

## 5. Conclusions

This work first confirmed previous studies showing gene expression alterations of oxidative stress, integrin-signaling, vesicle transportation, angiogenesis and vascular integrity in selected tissues of the *Pdcd10*-knockdown mouse model. Our findings reinforce the significance of these data and localize them in ECs, which are considered critical tissue for CMM formation. We also documented the involvement of novel pathways, including hypoxia, HIF-1α and Nod2 signaling, as well as immune response. Hopefully, these findings, if supported by further investigations and confirmed in other disease models, will contribute to the identification of a more personalized approach to disease prevention and treatment.

## Figures and Tables

**Table 1:** Differential expressed genes (Log Fold Change < 0.05).

Symbol	GeneID	Description	Ensembl_gene_id	Log Fold Change	*p*-Value	p-Adj
*Tnfrsf9*	21942	tumor necrosis factor receptor superfamily_ member 9	ENSMUSG00000028965	2.323	2.1 × 10^−28^	6.9 × 10^−24^
*Gbe1*	74185	glucan (1_4-alpha-)_ branching enzyme 1	ENSMUSG00000022707	2.149	6.6 × 10^−27^	1.0 × 10^−22^
*Arrb1*	109689	arrestin_ beta 1	ENSMUSG00000018909	2.000	4.6 × 10^−21^	5.1 × 10^−17^
*P4ha2*	18452	procollagen-proline_ 2-oxoglutarate 4-dioxygenase (proline 4-hydroxylase)_ alpha II polypeptide	ENSMUSG00000018906	1.529	1.8 × 10^−17^	1.5 × 10^−13^
*Vwa1*	246228	von Willebrand factor A domain containing 1	ENSMUSG00000042116	2.035	3.4 × 10^−16^	2.2 × 10^−12^
*Emc2*	66736	ER membrane protein complex subunit 2	ENSMUSG00000022337	−1.413	4.9 × 10^−16^	2.6 × 10^−12^
*Fam19a5*	106014	family with sequence similarity 19_ member A5	ENSMUSG00000054863	1.360	5.0 × 10^−11^	2.3 × 10^−7^
*Epm2a*	13853	epilepsy_ progressive myoclonic epilepsy_ type 2 gene alpha	ENSMUSG00000055493	1.590	9.7 × 10^−10^	4.0 × 10^−6^
*Csf2rb*	12983	colony stimulating factor 2 receptor_ beta_ low-affinity (granulocyte-macrophage)	ENSMUSG00000071713	1.763	1.7 × 10^−9^	6.2 × 10^−6^
*Hist2h2aa1*	15267	histone cluster 2_ H2aa1	ENSMUSG00000064220	1.484	3.1 × 10^−9^	1.0 × 10^−5^
*Lamb3*	16780	laminin_ beta 3	ENSMUSG00000026639	1.236	5.8 × 10^−9^	1.6 × 10^−5^
*Serpine1*	18787	serine (or cysteine) peptidase inhibitor_ clade E_ member 1	ENSMUSG00000037411	1.530	5.4 × 10^−9^	1.6 × 10^−5^
*Adam8*	11501	a disintegrin and metallopeptidase domain 8	ENSMUSG00000025473	1.679	6.3 × 10^−9^	1.6 × 10^−5^
*Ndrg1*	17988	N-myc downstream regulated gene 1	ENSMUSG00000005125	1.425	1.0 × 10^−8^	2.3 × 10^−5^
*Gipr*	381853	gastric inhibitory polypeptide receptor	ENSMUSG00000030406	1.711	1.2 × 10^−8^	2.8 × 10^−5^
*Tenm4*	23966	teneurin transmembrane protein 4	ENSMUSG00000048078	1.251	1.7 × 10^−8^	3.6 × 10^−5^
*Elp5*	54351	elongator acetyltransferase complex subunit 5	ENSMUSG00000018565	−0.881	3.0 × 10^−8^	5.3 × 10^−5^
*Ghrh*	14601	growth hormone releasing hormone	ENSMUSG00000027643	1.642	2.8 × 10^−8^	5.3 × 10^−5^
*Gm14253*	628707	programmed cell death 10 pseudogene	ENSMUSG00000082321	−1.498	3.0 × 10^−8^	5.3 × 10^−5^
*Nos2*	18126	nitric oxide synthase 2_ inducible	ENSMUSG00000020826	1.655	3.8 × 10^−8^	5.9 × 10^−5^
*Selenbp1*	20341	selenium binding protein 1	ENSMUSG00000068874	1.655	3.7 × 10^−8^	5.9 × 10^−5^
*Pak3*	18481	p21 protein (Cdc42/Rac)-activated kinase 3	ENSMUSG00000031284	−0.937	5.3 × 10^−8^	7.9 × 10^−5^
*Fstl3*	83554	follistatin-like 3	ENSMUSG00000020325	1.134	8.3 × 10^−8^	1.1 × 10^−4^
*Gm43552*	NULL	predicted gene 43552	ENSMUSG00000105835	1.585	8.4 × 10^−8^	1.1 × 10^−4^
*Pdcd10*	56426	programmed cell death 10	ENSMUSG00000027835	−1.188	1.0 × 10^−7^	1.3 × 10^−4^
*Itpk1*	217837	inositol 1_3_4-triphosphate 5/6 kinase	ENSMUSG00000057963	1.372	9.9 × 10^−8^	1.3 × 10^−4^
*Ero1l*	50527	ERO1-like (S. cerevisiae)	ENSMUSG00000021831	1.543	1.3 × 10^−7^	1.5 × 10^−4^
*Edil3*	13612	EGF-like repeats and discoidin I-like domains 3	ENSMUSG00000034488	1.537	1.4 × 10^−7^	1.6 × 10^−4^
*AU021092*	239691	expressed sequence AU021092	ENSMUSG00000051669	1.576	1.4 × 10^−7^	1.6 × 10^−4^
*Hmox1*	15368	heme oxygenase 1	ENSMUSG00000005413	1.109	1.6 × 10^−7^	1.7 × 10^−4^
*Erlin2*	244373	ER lipid raft associated 2	ENSMUSG00000031483	0.912	1.9 × 10^−7^	2.0 × 10^−4^
*Emilin2*	246707	elastin microfibril interfacer 2	ENSMUSG00000024053	0.960	2.3 × 10^−7^	2.3 × 10^−4^
*Aire*	11634	autoimmune regulator (autoimmune polyendocrinopathy candidiasis ectodermal dystrophy)	ENSMUSG00000000731	1.520	4.4 × 10^−7^	4.1 × 10^−4^
*Id2*	15902	inhibitor of DNA binding 2	ENSMUSG00000020644	1.091	4.2 × 10^−7^	4.1 × 10^−4^
*Tnnc1*	21924	troponin C_ cardiac/slow skeletal	ENSMUSG00000091898	−1.209	4.3 × 10^−7^	4.1 × 10^−4^
*Adm2*	223780	adrenomedullin 2	ENSMUSG00000054136	1.341	4.9 × 10^−7^	4.5 × 10^−4^
*Gas2l3*	237436	growth arrest-specific 2 like 3	ENSMUSG00000074802	−0.923	6.1 × 10^−7^	5.4 × 10^−4^
*Adgrb2*	230775	adhesion G protein-coupled receptor B2	ENSMUSG00000028782	−1.050	6.8 × 10^−7^	5.8 × 10^−4^
*Sema7a*	20361	sema domain_ immunoglobulin domain (Ig)_ and GPI membrane anchor_ (semaphorin) 7A	ENSMUSG00000038264	1.215	8.4 × 10^−7^	6.9 × 10^−4^
*Sbsn*	282619	suprabasin	ENSMUSG00000046056	1.041	8.4 × 10^−7^	6.9 × 10^−4^
*Ush2a*	22283	Usher syndrome 2A (autosomal recessive_ mild)	ENSMUSG00000026609	1.447	1.3 × 10^−6^	1.0 × 10^−3^
*Prr11*	270906	proline rich 11	ENSMUSG00000020493	−0.812	1.4 × 10^−6^	1.1 × 10^−3^
*Ostf1*	20409	osteoclast stimulating factor 1	ENSMUSG00000024725	0.933	1.9 × 10^−6^	1.5 × 10^−3^
*Timp3*	21859	tissue inhibitor of metalloproteinase 3	ENSMUSG00000020044	0.909	3.5 × 10^−6^	2.6 × 10^−3^
*Tnfrsf23*	79201	tumor necrosis factor receptor superfamily_ member 23	ENSMUSG00000037613	0.764	4.7 × 10^−6^	3.4 × 10^−3^
*Gys1*	14936	glycogen synthase 1_ muscle	ENSMUSG00000003865	1.075	5.1 × 10^−6^	3.6 × 10^−3^
*Sprr1a*	20753	small proline-rich protein 1A	ENSMUSG00000050359	1.348	5.9 × 10^−6^	4.1 × 10^−3^
*Dmkn*	73712	dermokine	ENSMUSG00000060962	1.088	6.8 × 10^−6^	4.7 × 10^−3^
*Plin4*	57435	perilipin 4	ENSMUSG00000002831	1.242	7.8 × 10^−6^	5.2 × 10^−3^
*Sparcl1*	13602	SPARC-like 1	ENSMUSG00000029309	1.025	8.1 × 10^−6^	5.2 × 10^−3^
*Mthfd1l*	270685	methylenetetrahydrofolate dehydrogenase (NADP+ dependent) 1-like	ENSMUSG00000040675	0.984	8.2 × 10^−6^	5.2 × 10^−3^
*Gcnt2*	14538	glucosaminyl (N-acetyl) transferase 2_ I-branching enzyme	ENSMUSG00000021360	0.778	8.7 × 10^−6^	5.5 × 10^−3^
*Acap1*	216859	ArfGAP with coiled-coil_ ankyrin repeat and PH domains 1	ENSMUSG00000001588	1.306	9.5 × 10^−6^	5.9 × 10^−3^
*Hist1h2bg*	319181	histone cluster 1_ H2bg	ENSMUSG00000058385	1.002	1.0 × 10^−5^	6.5 × 10^−3^
*Kctd10*	330171	potassium channel tetramerisation domain containing 10	ENSMUSG00000001098	−0.635	1.2 × 10^−5^	7.2 × 10^−3^
*Porcn*	53627	porcupine homolog (Drosophila)	ENSMUSG00000031169	1.051	1.2 × 10^−5^	7.2 × 10^−3^
*Fam162a*	70186	family with sequence similarity 162_ member A	ENSMUSG00000003955	0.872	1.4 × 10^−5^	8.2 × 10^−3^
*Arhgap19*	71085	Rho GTPase activating protein 19	ENSMUSG00000025154	−0.806	1.8 × 10^−5^	1.0 × 10^−2^
*Sept11*	52398	septin 11	ENSMUSG00000058013	−0.757	1.9 × 10^−5^	1.0 × 10^−2^
*Fdft1*	14137	farnesyl diphosphate farnesyl transferase 1	ENSMUSG00000021273	−0.834	1.9 × 10^−5^	1.0 × 10^−2^
*Foxc1*	17300	forkhead box C1	ENSMUSG00000050295	−0.687	2.0 × 10^−5^	1.1 × 10^−2^
*Car12*	76459	carbonic anyhydrase 12	ENSMUSG00000032373	1.240	2.0 × 10^−5^	1.1 × 10^−2^
*Fosl1*	14283	fos-like antigen 1	ENSMUSG00000024912	0.952	2.4 × 10^−5^	1.2 × 10^−2^
*Fstl1*	14314	follistatin-like 1	ENSMUSG00000022816	−0.903	2.6 × 10^−5^	1.3 × 10^−2^
*Mt1*	17748	metallothionein 1	ENSMUSG00000031765	1.264	2.6 × 10^−5^	1.3 × 10^−2^
*2610528A11Rik*	70045	RIKEN cDNA 2610528A11 gene	ENSMUSG00000096001	1.210	2.9 × 10^−5^	1.4 × 10^−2^
*Minpp1*	17330	multiple inositol polyphosphate histidine phosphatase 1	ENSMUSG00000024896	−0.857	2.9 × 10^−5^	1.4 × 10^−2^
*Lrig1*	16206	leucine-rich repeats and immunoglobulin-like domains 1	ENSMUSG00000030029	−0.607	3.2 × 10^−5^	1.5 × 10^−2^
*L1cam*	16728	L1 cell adhesion molecule	ENSMUSG00000031391	1.072	3.4 × 10^−5^	1.6 × 10^−2^
*Ttll12*	223723	tubulin tyrosine ligase-like family_ member 12	ENSMUSG00000016757	−0.752	3.9 × 10^−5^	1.8 × 10^−2^
*Gpr35*	64095	G protein-coupled receptor 35	ENSMUSG00000026271	1.193	3.8 × 10^−5^	1.8 × 10^−2^
*Gm14760*	654474	glyceraldehyde-3-phosphate dehydrogenase pseudogene	ENSMUSG00000081221	1.204	3.9 × 10^−5^	1.8 × 10^−2^
*Nde1*	67203	nuclear distribution gene E homolog 1 (A nidulans)	ENSMUSG00000022678	−0.786	4.3 × 10^−5^	1.9 × 10^−2^
*4930447F24Rik*	76873	RIKEN cDNA 4930447F24 gene	ENSMUSG00000102224	0.976	4.2 × 10^−5^	1.9 × 10^−2^
*Csf2rb2*	12984	colony stimulating factor 2 receptor_ beta 2_ low-affinity (granulocyte-macrophage)	ENSMUSG00000071714	1.227	4.4 × 10^−5^	1.9 × 10^−2^
*Ankrd37*	654824	ankyrin repeat domain 37	ENSMUSG00000050914	1.045	4.5 × 10^−5^	1.9 × 10^−2^
*Ola1*	67059	Obg-like ATPase 1	ENSMUSG00000027108	−0.598	4.8 × 10^−5^	2.0 × 10^−2^
*Gm43951*	NULL	predicted gene_ 43951	ENSMUSG00000107877	1.208	4.8 × 10^−5^	2.0 × 10^−2^
*Plxnd1*	67784	plexin D1	ENSMUSG00000030123	0.683	5.3 × 10^−5^	2.2 × 10^−2^
*Foxred2*	239554	FAD-dependent oxidoreductase domain containing 2	ENSMUSG00000016552	0.650	5.6 × 10^−5^	2.3 × 10^−2^
*9330151L19Rik*	414085	RIKEN cDNA 9330151L19 gene	ENSMUSG00000097061	0.937	6.3 × 10^−5^	2.5 × 10^−2^
*Rac3*	170758	RAS-related C3 botulinum substrate 3	ENSMUSG00000018012	0.831	6.7 × 10^−5^	2.6 × 10^−2^
*Dusp5*	240672	dual specificity phosphatase 5	ENSMUSG00000034765	0.773	6.7 × 10^−5^	2.6 × 10^−2^
*Arl6ip5*	65106	ADP-ribosylation factor-like 6 interacting protein 5	ENSMUSG00000035199	0.665	6.9 × 10^−5^	2.7 × 10^−2^
*Sort1*	20661	sortilin 1	ENSMUSG00000068747	−0.887	7.1 × 10^−5^	2.7 × 10^−2^
*Gm3200*	100041204	glyceraldehyde-3-phosphate dehydrogenase pseudogene	ENSMUSG00000097388	1.118	7.1 × 10^−5^	2.7 × 10^−2^
*Aldh3a2*	11671	aldehyde dehydrogenase family 3_ subfamily A2	ENSMUSG00000010025	−0.725	7.3 × 10^−5^	2.7 × 10^−2^
*Slc2a1*	20525	solute carrier family 2 (facilitated glucose transporter)_ member 1	ENSMUSG00000028645	0.851	7.7 × 10^−5^	2.8 × 10^−2^
*Tusc2*	80385	tumor suppressor candidate 2	ENSMUSG00000010054	0.675	8.5 × 10^−5^	3.1 × 10^−2^
*M6pr*	17113	mannose-6-phosphate receptor_ cation dependent	ENSMUSG00000007458	0.610	8.9 × 10^−5^	3.2 × 10^−2^
*Pfkp*	56421	phosphofructokinase_ platelet	ENSMUSG00000021196	0.844	8.9 × 10^−5^	3.2 × 10^−2^
*Cda*	72269	cytidine deaminase	ENSMUSG00000028755	0.684	1.0 × 10^−4^	3.8 × 10^−2^
*Eef2k*	13631	eukaryotic elongation factor-2 kinase	ENSMUSG00000035064	0.624	1.0 × 10^−4^	3.8 × 10^−2^
*Ogn*	18295	osteoglycin	ENSMUSG00000021390	−0.989	1.4 × 10^−4^	4.9 × 10^−2^

**Table 2:** Enriched significant pathways (FDR-adjusted *p*-Value ≤ 0.05). FOOTNOTE: N.: number.

Term Name	*p*-Value	FDR-Adjusted *p*-Value	N. of Genes that Are Also in This Filter or Cluster	N. of Upregulated Genes	N. of Downregulated Genes
HYPOXIA	8.2 × 10^−9^	3.0 × 10^−7^	13	13	0
HIF-1-α transcription factor network	7.4 × 10^−6^	0.00020	6	6	0
GSE21360_SECONDARY_VS_QUATERNARY_MEMORY_CD8_TCELL_UP	3.8 × 10^−7^	0.00071	9	9	0
GSE3982_DC_VS_BCELL_UP	4.1 × 10^−7^	0.00071	9	8	1
GSE21360_SECONDARY_VS_QUATERNARY_MEMORY_CD8_TCELL_DN	1.8 × 10^−6^	0.00208	8	8	0
GSE22611_NOD2_TRANSDUCED_VS_CTRL_HEK293T_STIMULATED_WITH_MDP_2H_DN	5.1 × 10^−6^	0.00352	8	8	0
GSE37416_CTRL_VS_6H_F_TULARENSIS_LVS_NEUTROPHIL_DN	3.9 × 10^−5^	0.01260	7	7	0
GSE360_CTRL_VS_L_DONOVANI_DC_DN	3.3 × 10^−5^	0.01260	7	7	0
GSE15930_NAIVE_VS_72H_IN_VITRO_STIM_IL12_CD8_TCELL_DN	6.4 × 10^−5^	0.01271	7	5	2
Glycogen biosynthesis II (from UDP-D-Glucose)	0.00083	0.01995	2	2	0
ESTROGEN_RESPONSE_LATE	0.00201	0.03626	7	3	4
GSE25677_MPL_VS_R848_STIM_BCELL_UP	0.00031	0.04286	6	6	0
GSE36078_UNTREATED_VS_AD5_INF_MOUSE_LUNG_DC_UP	0.00043	0.04286	6	5	1
GSE43955_1H_VS_42H_ACT_CD4_TCELL_WITH_TGFB_IL6_DN	0.00044	0.04286	6	6	0
GSE17721_CpG_VS_GARDIQUIMOD_1H_BMDC_UP	0.00042	0.04286	6	4	2
GSE3982_MAC_VS_BCELL_UP	0.00039	0.04286	6	6	0
GSE17301_CTRL_VS_48H_ACD3_ACD28_IFNA2_STIM_CD8_TCELL_UP	0.00040	0.04286	6	4	2
EPITHELIAL_MESENCHYMAL_TRANSITION	0.00620	0.05306	6	5	1
ESTROGEN_RESPONSE_EARLY	0.00722	0.05306	6	3	3
TNFA_SIGNALING_VIA_NFKB	0.00758	0.05306	6	6	0

**Table 3:** List of significant genes associated with Hypoxia and HFI-1 pathways.

Pathway	Gene	Description	Aliases
**Hypoxia**	*Gbe1*	glucan (1,4-alpha), branching enzyme 1	2310045H19RIK, 2810426P10RIK, D16ERTD536E
	*Serpine1*	serine (or cysteine) peptidase inhibitor, clade E, member 1	PAI-1, PAI1, PLANH1
	*Car12*	carbonic anhydrase 12	2310047E01RIK, AI314958, CA-XII, CA12
	*Fam162a*	family with sequence similarity 162, member A	2310056P07RIK, HGTD-P
	*Gcnt2*	glucosaminyl (N-acetyl) transferase 2, I-branching enzyme	5330430K10RIK, IGNT, IGNTA, IGNTB, IGNTC
	*Selenbp1*	selenium binding protein 1	LP56, LPSB, SBP56
	*P4ha2*	procollagen-proline, 2-oxoglutarate 4-dioxygenase (proline 4-hydroxylase), alphaII polypeptide	AA407196, C76437, P4HL
	*Hmox1*	heme oxygenase 1	D8WSU38E, HO-1, HO1, HEMOX, HMOX, HSP32
	*Slc2a1*	solute carrier family 2 (facilitated glucose transporter), member 1	GLUT-1, GLUT1
	*Ero1l*	ERO1-like (S. cerevisiae)	ERO1-L
	*Ndrg1*	N-myc downstream-regulated gene 1	CAP43, CMT4D, DRG1, HMSNL, NMSL, NDR1, NDRL, PROXY1, RTP, TDD5
	*Gys1*	glycogen synthase 1, muscle	GYS3, MGS
	*Pfkp*	phosphofructokinase, platelet	1200015H23RIK, 9330125N24RIK, ATP-PFK, PFK-C, PFK-P
**HIF-1-alpha**	*Nos2*	nitric oxide synthase 2, inducible	MAC-NOS, NOS-II, NOS-2, NOS2A, I-NOS, INOS
	*Hmox1*	heme oxygenase 1	D8WSU38E, HO-1, HO1, HEMOX, HMOX, HSP32
	*Serpine1*	serine (or cysteine) peptidase inhibitor, clade E, member 1	PAI-1, PAI1, PLANH1
	*Id2*	inhibitor of DNA binding 2	AI255428, C78922, IDB2, BHLHB26
	*Ndrg1*	N-myc downstream-regulated gene 1	CAP43, CMT4D, DRG1, HMSNL, NMSL, NDR1, NDRL, PROXY1, RTP, TDD5
	*Slc2a1*	solute carrier family 2 (facilitated glucose transporter), member 1	GLUT-1, GLUT1

## Data Availability

Not applicable.

## Supplementary Materials

The following supporting information can be downloaded at: https://www.mdpi.com/article/10.3390/genes13060961/s1, Figure S1: immunofluorescence analyses of PECAM 1 protein; Table S1: sequences of mouse primers used in this study for qRT-PCR study; Table S2: all differential expressed genes; Table S2: biological process annotation clustering by different databases.
