# Supplementary material for: Transcriptome Analysis Reveals Altered Expression of Genes Involved in Hypoxia, Inflammation and Immune Regulation in Pdcd10-Depleted Mouse Endothelial Cells
Source: Genes (Basel). 2022 May 27;13(6):961. doi: 10.3390/genes13060961 (PMC9222422; doi:10.3390/genes13060961)
Supplement: Supplementary file 1 [file genes-13-00961-s001.zip › Table S1.pdf]

Table S1. Sequences of mouse primers used in this study for qRT-PCR study.

Gene Name	Ensembl Gene ID	Primer pair
Adam8	**ENSMUSG00000025473**	mAdam8-RT-F: GGTATTGCAGGGCACCAAGT
		mAdam8-RT-R: CCTGGCAGCTTCCATCCAT
Csf2rb	**ENSMUSG00000071713**	mCsf2rb-RT-F: TTTCCTTTGGGCTCTTCTATCG
		mCsf2rb-RT-R: CACCACCGGAGAGCATTTCT
Fam162a	**ENSMUSG00000003955**	mFam162a-RT-F: TGCACCAAGCCACAGGAAA
		mFam162a-RT-R: GGCACCCCGTGTCTGTAAGA
Fosl1	**ENSMUSG00000024912**	mFosl1-RT-F: CCGAAGAAAGGAGCTGACAGA
		mFosl1-RT-R: CGATTTCTCATCCTCCAATTTGT
Gbe1	**ENSMUSG00000022707**	mGbe1-RT-F: CCTTCGCCGCGGACTT
		mGbe1-RT-R: AATGTCATGCAAAACCTGGCTAA
Ghrh	**ENSMUSG00000027643**	mGhrh-RT-F: GCTGTATGCCCGGAAAGTGA
		mGhrh-RT-R: TGGATCCTCTCCCCTTGCT
Gys1	**ENSMUSG00000003865**	mGys1-RT_F: AGAGAGCCATCTTTGCCACTCA
		mGys1-RT_R: GCATGTTGTGGGTGCACACT
Hist1h2bg	**ENSMUST00000079251**	mHist1h2bg-RT-F: CACCGGCATCTCCTCCAA
		mHist1h2bg-RT-R: CGCTCGAAGATGTCGTTCAC
Hmox1	**ENSMUSG00000005413**	mHmox1-RT-F: CAGGTGTCCAGAGAAGGCTTTAA
		mHmox1-RT-R: CAGGGCCGTGTAGATATGGTACA
Mthfd1l	**ENSMUSG00000040675**	mMthfd1l-RT-F: GCCCCCGGGATGGA
		mMthfd1l-RT-R: AGCTCAGCACTTCCTTGGAATT
Ndrg1	**ENSMUSG00000005125**	mNdrg1-RT-F : GCTTGCTGAGGCCTTCAAGT
		mNdrg1-RT-R:TGCTGGCAGAAGGCATGTATC
Nos2	**ENSMUSG00000020826**	mNos2-RT-F : CCCCGCAGCTCCTCACT
		mNos2-RT-R:TGCAGCTTGTCCAGGGATTC
P4ha2	**ENSMUSG00000018906**	mP4ha2-RT-F: CTGGGTGCAGGCAGAATTCT
		mP4ha2-RT-R TGCGTAAATCAGATCGGTCATG
Pfkp	**ENSMUSG00000021196**	mPfkp-RT_F: ATGGGACTGTGTCTCCAGCAT
		mPfkp-RT_R: CGCGCACTACCGATGATG
Serpine1	**ENSMUSG00000037411**	mSerpine1-RT-F: GCCCCGCCTCCTCATC
		mSerpine1-RT-R: GCCCTCTGAGGTCCACTTCA
Slc2a1	**ENSMUSG00000028645**	mSlc2a1-RT-F : CGCCCCCCAGAAGGTTAT
		mSlc2a1-RT-R :TCCGTAGCGGTGGTTCCAT
Sort1	**ENSMUSG00000068747**	mSort1-RT-F: CCGCCTCCCGGACTTC
		mSort1-RT-R: AAAGACATGCTGGTGCGTATTGT
