# Supplementary figures and images for: Transcriptome Analysis Reveals Altered Expression of Genes Involved in Hypoxia, Inflammation and Immune Regulation in Pdcd10-Depleted Mouse Endothelial Cells
Source: Genes (Basel). 2022 May 27;13(6):961. doi: 10.3390/genes13060961 (PMC9222422; doi:10.3390/genes13060961)

Figure S1

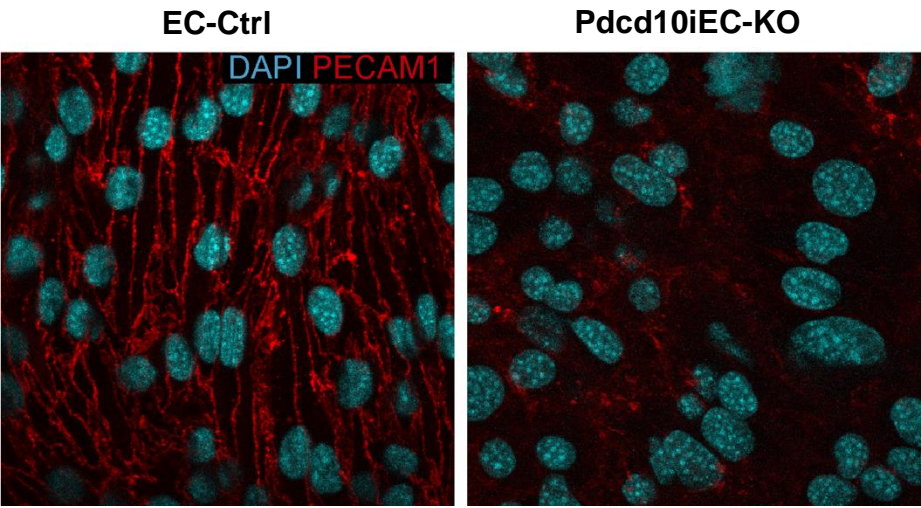

**Figure S1:** immunofluorescence analyses of PECAM1 protein in EC-Ctrl and *Pdc10iEC-KO* cells.

Supplement: Supplementary file 1 [file genes-13-00961-s001.zip › Figure S1.pdf]
